# While they wait: a cross-sectional survey on wait times for mental health treatment for anxiety and depression for adolescents in Australia

**DOI:** 10.1136/bmjopen-2024-087342

**Published:** 2025-03-24

**Authors:** Mirjana Subotic-Kerry, Thomas Borchard, Belinda Parker, Sophie H Li, Jayden Choi, Emma V Long, Philip J Batterham, Alexis Whitton, Aniela Gockiert, Lucinda Spencer, Bridianne O’Dea

**Affiliations:** 1 University of New South Wales Faculty of Medicine, Sydney, New South Wales, Australia; 2 Black Dog Institute, Randwick, New South Wales, Australia; 3 ANROWS, Sydney, New South Wales, Australia; 4 Centre for Mental Health Research, Research School of Population Health, Australian National University, Canberra, Australian Capital Territory, Australia; 5 School of Psychology, Western Sydney University, Penrith, New South Wales, Australia; 6 Grand Pacific Health, Wollongong, New South Wales, Australia; 7 Flinders University Institute for Mental Health and Wellbeing, Adelaide, South Australia, Australia

**Keywords:** MENTAL HEALTH, Adolescents, Anxiety disorders, Depression & mood disorders, Waiting lists

## Abstract

**Abstract:**

**Background:**

Long wait times impede timely access to mental health treatment for anxiety and depression for adolescents. However, there is limited quantitative research on current wait times for the treatment of anxiety and depression for adolescents in Australia and the impact of wait times on adolescent help-seekers.

**Aims:**

This study examined adolescents’ experiences of wait times for the treatment of anxiety and depression in Australia, including the providers they were waiting to access, the self-reported duration and perceived acceptability of wait times, the association between these wait times and psychological distress and the support and coping behaviours used by adolescents during this time.

**Method:**

From April to June 2022, 375 adolescents aged 13–17 years who were living in Australia and currently waiting, or had previously waited in the past 12 months, for mental health treatment for anxiety and depression completed a cross-sectional online survey.

**Results:**

Most adolescents initiated care with psychologists and psychiatrists, with mean wait times of 100.1 days (SD: 77.25) and 127.5 days (SD: 78.80), respectively. The mean wait time across all treatment providers was 99.6 days (SD: 80.44). Most participants (85.2%) felt their wait times were ‘too long’. Longer wait times were associated with increased psychological distress, and many adolescents perceived that their mental health worsened during the wait time. Most participants did not receive any support from their healthcare providers during the wait time and engaged in maladaptive and risky coping behaviours while waiting. However, self-reported treatment attendance remained high.

**Conclusions:**

Adolescents in Australia face lengthy wait times when accessing mental health treatment, and this may exacerbate distress and maladaptive coping.

**STRENGTHS AND LIMITATIONS OF THIS STUDY**

- By examining various dimensions of wait times, including duration, perceived acceptability and impacts on mental health, the study provides a comprehensive understanding of wait times for mental health services for anxiety and depression.
- The survey used in this study was developed in consultation with young people, mental health professionals and researchers and covered a broad spectrum of experiences regarding wait times for mental health services.
- The recruitment strategy was broad, using social media and partnerships with clinical services to reach youth from all states and territories within Australia.
- The cross-sectional nature of the study limits the ability to determine causal relationships between wait times and mental health outcomes.
- Participants who are more engaged or have stronger opinions about their wait times might have been more likely to participate, and we may not have captured the views of adolescents who attended their first treatment session within a short time frame or who were satisfied with their wait time.

## Introduction

### Wait times for adolescent mental health services in Australia

 Anxiety and depression are common mental health problems among adolescents in Australia and worldwide.^1 2^ Although effective treatments exist, long wait times impede access to mental health services and are a major barrier to treatment uptake among youth.^3^,^5^ While wait times for mental health treatment vary across countries^4^,^6^ and services,^7 8^ the increasing demand for treatment coupled with the COVID-19 pandemic has placed increased pressure on mental health systems globally.^9 10^ Prior to the pandemic, the headspace public youth mental health service in Australia reported an average wait of 25.5 days for psychological treatment,^3^ whereas a secret shopper study of Australian psychologists and psychiatrists reported a median wait time of 34 days and 41 days, respectively.^11^ During the pandemic, 88% of psychologists in Australia reported that their wait times had increased, with one in two clients waiting more than 3 months for their first session of treatment.^12^ Similar patterns have been reported in the USA, UK, Canada and other countries;^4^,^68^ however, the current wait times for mental health treatment in Australia and the impacts of these on adolescents are unclear.

### The impact of wait times on adolescents’ mental health

Evidence is emerging on the potential negative consequences of extended wait times on young people’s mental health and treatment uptake. The waiting time between referral and treatment provision has been identified as a period of significant vulnerability for adolescents and their families, as individuals’ symptoms are acute, but treatment has not yet begun. Prolonged wait times are associated with the premature termination of treatment,^13^ lower rates of kept appointments^14^ and increased number of missed appointments.^13 15 16^ Research has also found that longer wait times are associated with symptom deterioration and diminished future help-seeking,^17^ with qualitative reports of increased negative emotional and behavioural consequences and worsened psychological health.^18^ Despite these potential negative impacts, there is a scarcity of quantitative data on wait times for adolescent mental health treatment in Australia.

### Wait time standards for mental health treatment

A key hallmark of high-performing mental health systems is the timely accessibility and availability of treatment services.^19^ In many countries, national waiting time standards for mental health treatment have been introduced to monitor the performance of mental healthcare systems.^19^ In 2016, the National Health Service (NHS) in the UK established wait time targets with 75% of referrals for psychological interventions for anxiety and depression to begin treatment within 6 weeks and 95% within 18 weeks.^20 21^ This performance benchmarking was found to significantly reduce wait times, with over 90% of referrals having accessed care within 6 weeks.^22^ The NHS standards have since been updated to include a 4-week wait time target for children and young people.^23^ This is consistent with Norway, where the national wait time target for youth mental healthcare is 35 days.^24^ There are currently no national efforts to collect or benchmark the wait times for mental health services in Australia using transparent methods. As such, our knowledge of adolescents’ experiences of wait times in Australia is limited.

### Objectives of the current study

The current study aimed to explore adolescents’ (aged 13–17) experiences of wait times for mental health treatment for depression and anxiety in Australia. This study examined service utilisation, self-reported wait time duration and perceived acceptability of wait times among adolescents seeking treatment for depression or anxiety. The associations between self-reported wait times and adolescents’ psychological distress, as well as any perceived changes in mental health experienced by young people during their wait time, were also examined. Lastly, this study explored the support adolescents received during their wait time, the coping behaviours they used while awaiting care, and their self-reported treatment attendance.

Based on past studies, it was hypothesised that treatment-seeking adolescents in Australia with depression and anxiety would report an average wait time of at least 1 month for mental health treatment and services.^3 11^ It was also hypothesised that longer wait times would be associated with greater levels of psychological distress. To our knowledge, this is one of the first studies to examine this aspect of mental healthcare service provision for adolescents in Australia and provides much-needed insight on how to better support young people as they await care.

## Method

### Design

An online cross-sectional survey was administered between April and June 2022. The Black Dog Institute’s Youth Lived Experience Advisory Group was consulted on all aspects of the study design.

### Patient and public involvement

The survey was written for this study in consultation with young people, mental health professionals and researchers. See online supplemental material for a detailed description of the survey development and online supplemental appendix A for the full survey including all response options.

###  Ethical approval

The authors assert that all procedures contributing to this work comply with the ethical standards of the relevant national and institutional committees on human experimentation and with the Helsinki Declaration of 1975, as revised in 2008.

### Sample size

The target sample size was 383 participants based on a confidence level of 95%, a population size of n=97 500^1^ and a margin of error of 5%. The population size reflects the estimated number of adolescents in Australia aged 13–17 years who meet the criteria for a clinical diagnosis of anxiety and/or depression and are likely to seek mental health treatment based on a nationally representative sample.^1^

### Participants

Adolescents were eligible to participate if they were aged 13–17 years old living in Australia and had sought treatment for anxiety and/or depression in the past 12 months. To enable greater exploration of wait times and participation among adolescents, we included two subgroups of participants: (1) adolescents who were currently waiting to attend their first-ever session of mental health treatment and (2) adolescents who had waited more than 1 week in the past 12 months to access their first-ever session of treatment. Adolescents were excluded if they were (1) currently waiting for a follow-up treatment session with a mental health professional or service that they were not accessing for the first time, or (2) currently waiting or previously waiting for a treatment session that was unrelated to anxiety or depression.

### Recruitment, procedure and consent

Participants were recruited via paid social media campaigns on Facebook, Twitter, Instagram and LinkedIn (for parents and carers to promote to youth). Study information was also published on the research sponsor’s (Black Dog Institute) website and circulated through their clinical service partners. The Black Dog Institute is a mental health research institute in Australia affiliated with the University of New South Wales (UNSW). The Institute’s website promotes research participation opportunities to a range of diverse audiences. All recruitment materials were submitted and approved by the UNSW Human Research Ethics Committee. All study advertisements provided hyperlinks to the survey.

Before commencing the survey, participants were presented with the participant information sheet and were required to pass screening questions and a 4-item Gillick Competence Test.^25^ This test was used to measure the capacity of adolescents aged under 18 years to provide informed consent to participate in research. Four questions, answered using three multiple choice options, tested the participant’s comprehension of what the research study involved (‘This research study involves…’), who the research study was being conducted by (‘This research is being conducted by…’), the voluntary nature of participation (‘Do I have to finish the survey?’) and who their responses would be shared with (‘Your responses to this survey will be shared with…’). Individuals who did not complete the items correctly were excluded. For a full copy, please see online supplemental appendix A.

Active parental consent was not obtained in the current study due to the use of a Gillick Competence measure, the anonymous nature of the survey, and the minimal risk of harm from a young person’s involvement. The survey provided all participants with information on Australia-based help-seeking resources. All eligible individuals provided consent via an online form, and all participants who completed the survey received a $A20 voucher sent via email.

### Survey measures

#### Demographics

Participants were asked to report their age, gender identity, whether they identified as Aboriginal and/or Torres Strait Islander, whether they identified as Lesbian, Gay, Bisexual, Trans, Queer, Intersex, Asexual or another diverse sexual identity (LGBTQIA+), the Australian State or Territory and postcode they were currently living in and their educational/employment status. Postcodes were then classified as ‘metropolitan’ or ‘non-metropolitan’ according to the Australian Bureau of Statistics 2016 Australian Statistical Geography Standard.^26^

#### History of mental health

Participants were asked whether they had ever been formally diagnosed with depression and/or anxiety by a health professional and whether they were currently taking medication prescribed by a health professional for depression and/or anxiety.

#### Treatment providers, wait time duration, perceived acceptability of wait time

Participants were asked to review a list of 11 mental health treatment providers and indicate which professionals and services they were currently waiting to see for the first time (ie, professionals and services they had been referred to, contacted and made an appointment with). These included a psychologist, psychiatrist, headspace centre, hospital stay, a programme or service to help improve feelings of sadness or worry (eg, Cool Kids), Local Child and Adolescent Mental Health Service (CAHMS), school counsellor, paediatrician, a support group (eg, a group of people meeting to share information, experiences, problems and solutions), an Aboriginal/Torres Strait Islander medical centre, an Aboriginal/Torres Strait Islander support worker. There was a free response ‘other’ option included to list a professional or service that was not provided. For each of the treatment providers endorsed, participants were asked to report who referred them, the length of time waited between their first contact and attending their first session (how many months, weeks, days, or I don’t know/I can’t remember) and their perception of the wait time (too long, just right/acceptable, or unsure/I don’t know).

#### Psychological distress

Psychological distress was measured by the five-item Distress Questionnaire-5 (DQ5).^27^ Participants were asked to indicate the frequency with which they had experienced various thoughts, feelings and behaviours in the past 30 days from ‘never’ (1) to ‘always’ (5). Total scores range from 5 to 25 with higher scores indicating greater psychological distress, and a threshold of ≥14 as the clinical cut-off. This scale has demonstrated high internal consistency and convergent validity^27 28^ and has been used in adolescents.^29^ In the current study, the Cronbach’s alpha for the DQ5 was α=0.77.

#### Perceived changes in mental health during the wait time

Participants were asked to rate whether their feelings of sadness or worry had improved or worsened during their wait time using a 5-point Likert scale ranging from ‘worse’ (1) to ‘no change’ (3) to ‘better’ (5). Participants also had the option to select ‘does not apply to me’.

#### Support from healthcare providers during the wait time

Using a 5-point Likert scale ranging from ‘not at all important’ (1) to ‘extremely important’ (5), participants were asked to rate how important it was that their healthcare providers helped them manage their depression and anxiety while they awaited their first treatment session. Participants were then asked to rate how supported they felt by their healthcare providers while they awaited treatment using a 5-point Likert scale ranging from ‘not at all supported’ (1) to ‘extremely supported’ (5). Participants were then asked to report whether they had received any of the commonly provided resources during their wait time (eg, follow-up session or phone call with a general practitioner (GP), contact from the referred professional, information brochures on mental health and other support services). Two free response questions were asked: ‘Is there anything that your healthcare providers could have done to better support you during the wait time?’ and ‘What do you think would have helped you the most during your wait time?’.

#### Sources of personal support during the wait time

Participants were provided with a list of 17 sources of personal support and asked to rate how helpful each source was for them during the wait time. These included parents, siblings, other relatives of family members, friends, teacher, year advisor, school counsellor, other adult (eg, sports coach, a friend’s parent, a person at work), GP/local doctor, mental health professional (eg, psychologist, psychiatrist), telephone helplines (eg, Kids helpline, Lifeline), mental health websites (eg, ReachOut, Beyond Blue), online self-help mental health programmes (eg, programmes designed to help improve symptoms of sadness or worry), online assessment tools (eg, tools that ask you questions and tell you whether you are experiencing anxiety and/or depression), online support groups or discussion forums, online mental health chat services (eg, eHeadspace), mobile app for mental health. Responses were given using a 5-point Likert scale ranging from ‘not at all helpful (1)’ to ‘extremely helpful (5)’, with an additional option of ‘I didn’t seek/receive help from this source’. Participants were able to indicate other sources of support using a free response option.

#### Importance of additional support for parents/guardians during the wait time

Using a 5-point Likert scale ranging from ‘not at all’ (1) to ‘extremely’ (5), participants were asked to rate the importance of providing their parents or guardians with support to help them cope better during the wait time.

#### Coping behaviours used during the wait time

Participants were asked to select from a list of 26 randomly displayed behaviours that they had used to cope during their wait time. Participants could select all that applied. For analysis, each behaviour was collapsed into one of four categories: maladaptive (eg, spending more time online gaming), risky (eg, self-harming), help-seeking (eg, seeking support from friends), adaptive (eg, doing more exercise or sport). A free response option was also provided so that participants could report any coping behaviours that were not listed.

#### Attendance at first session of mental treatment

Participants who were currently waiting to access mental health treatment were asked how likely they were to attend their first session of treatment using a 5-point Likert scale ranging from ‘extremely unlikely’ (1) to ‘extremely likely’ (5). Participants who selected unlikely or extremely unlikely were then provided with a list of 11 reasons for non-attendance and were asked to select all that applied. Reasons for non-attendance included, I don’t need it anymore because I feel better, I found an earlier session somewhere else, I have had to wait for too long, I can’t be bothered, I might forget, I don’t have the money, I don’t want to go, The session is too far away from me, I don’t have any transport to get there, I feel too worried and/or sad to go, I am unsatisfied with the service, A different reason (please tell us in the text box).

Participants who had previously waited in the past 12 months to access mental health treatment were asked whether they attended their first session (‘yes’, ‘no’). Participants who reported that they did not attend were also provided with the same list of reasons for non-attendance as above and asked to select all that applied.

### Data analyses

Data were collected using Qualtrics and then exported to SPSS V.28.0^30^ for analysis. A detailed description of data cleaning processes is presented in the online supplemental material.

Researchers reviewed suspected fraudulent responses, and discrepancies were resolved by a third rater (see online supplemental material for additional information). Fraudulent and duplicate responses were detected by comparing participants’ details (email, postcode, IP addresses) and response patterns across the survey (see online supplemental table 1). Participants who completed the survey faster than 40% of the average completion time for the entire sample were removed as recommended by Cobanoglu *et al*.^31^

To determine wait time durations for treatment, the total mean days waited for each professional or service were calculated using the formula Total Months×30.437+Total Weeks×7+Total days waited. Any reported values above 1 year (365 days) for participants who were currently waiting to access their first-ever session of mental health treatment or 1 week or below for participants who had waited more than 1 week in the past 12 months to access their first session were removed. A total of 23 responses above 1 year and 40 values below or equal to 1 week were removed from the analysis.

Differences in wait times between metropolitan and regional/rural areas were examined using Mann-Whitney U tests. To compare wait times against the NHS benchmarks, the total days waited were collapsed into three categories: within 6 weeks (>1 week-42 days), within 18 weeks (>1 week–126 days) and greater than 18 weeks (127+days). To determine the association between wait times and psychological distress (DQ-5), zero-order correlations were conducted for those currently waiting only.

Qualitative (free response) data were analysed using Clarke and Braun’s^32^ six-stage thematic analysis guidelines, which allow for identifying and interpreting patterns of meaning within data.^33^ Given these questions were open-ended, an inductive approach was used to develop a coding framework.^34 35^ The analysis involved an iterative process of reading and coding responses and then organising codes into broader themes. Two primary coders (TB and EVL) independently coded a subset of responses for each free response question to create a preliminary framework, resolving discrepancies through discussion. The revised framework for each free response question was then applied to all responses, and codes were compared for consistency. Any discrepancies were resolved by a third independent rater (MS-K), ensuring consistency in code descriptions.

## Results

### Participants

A total of 780 respondents were assessed for study eligibility, and 92 were excluded due to being ineligible to participate (n=40) or failing the Gillick Competence Test (n=52). A further 313 responses were excluded due to being judged as invalid/fraudulent (n=211), incomplete (n=82) or completed too quickly (n=20) (see online supplemental figure 1).

The final sample consisted of 375 full completers (64.0% female, mean age: 16.04 years, SD=1.07, range: 13–17). For additional information, please refer to the online supplemental material. A total of 43.7% of the final sample (n=164/375) were currently waiting for their first session of mental treatment, and 56.3% (n=211/375) had previously waited, in the past 12 months, longer than 1 week to access their first treatment session. As shown in table 1, over half of the sample identified as being LGBTQIA+ (n=207/375; 55.2%). The majority lived in metropolitan areas (n=264/375; 70.4%) and were secondary school students (n=318/375; 84.8%). More than three-quarters of participants reported that they had received a formal diagnosis of depression and/or anxiety from a health professional (n=292/375; 77.9%) and 46.7% (n=175/375) reported that they were taking prescribed medication for their mental health.

**Table 1:** Participant demographics (n=375)

	N	%
Gender		
Male	67	17.9
Female	240	64.0
Non-binary	51	13.6
Different identity	14	3.7
I’d rather not say	3	0.8
Identified as Aboriginal/Torres Strait Islander peoples		
Aboriginal peoples	31	8.3
Torres Strait Islander peoples	1	0.3
Aboriginal and Torres Strait Islander peoples	1	0.3
Identified as LGBTQIA+	207	55.2
Metropolitan location	264	70.4
State or territory of residence		
Australian Capital Territory	5	1.3
New South Wales	107	28.5
Victoria	100	26.7
Queensland	82	21.9
Tasmania	22	5.9
Northern Territory	3	0.8
South Australia	29	7.7
Western Australia	27	7.2
Current education or employment status		
Secondary school	318	84.8
University	16	4.3
Apprenticeship/trade/full-time employment	12	3.2
Other	29	7.7
Formal diagnosis of depression and/or anxiety	292	77.9
Prescribed medication use for depression and/or anxiety	175	46.7

LGBTQIA+Lesbian, Gay, Bisexual, Transgender, Queer, Intersex, Asexual or another diverse sexual identity

### Treatment providers, wait time duration and perceived acceptability of wait times

Participants had initiated appointments with an average of 2.29 (SD: 1.31, range: 1–9) treatment providers, with psychologists (n=272; 72.5%) and psychiatrists (n=160; 42.7%) the most common (See table 2). Most participants (n=305/432, 70.6%) accessing psychologists and psychiatrists were referred by a GP. The average wait time across all treatment providers was 99.6 days (SD: 80.44, range: 8–365, median: 84.0). Please see table 2 for the mean and median wait times for each service provider. As shown, average and median wait times for the common treatment providers (ie, psychologists and psychiatrists) exceeded 3 months. However, there was significant variability in wait times within and across service providers, as demonstrated by the SD estimates ranging from 21.5 days to 89.4 days. Medical specialists (psychiatrists, paediatricians) were found to have the longest average wait times (127.5 days and 121.9 days, respectively), whereas services designed for acute and severe cases (CAMHS, inpatient units) and indigenous-specific services had the lowest wait times (71.6 days, 82.5 days and 45.7 days, respectively). The wait time to access a psychiatrist was significantly longer in metropolitan areas compared with regional areas (U=925.50, p*=*0.002). In contrast, the wait time was significantly longer in regional areas compared with metropolitan areas to access a paediatrician (U=63.50, p*=*0.042) and a school counsellor (U=347.50, p*=*0.014). All other comparisons by location did not reach significance (p=0.500–0.933). Across all treatment providers, most participants (n=548/643, 85.2%) perceived that their wait time was ‘too long’.

**Table 2:** Treatment providers, wait time durations and perceived acceptability of wait times among participants (n=375)

Treatment providers	N (%) utilising this service	GP referred N (%)	N who reported wait time	Mean days waited (SD)	Median days waited	Range (days)	N (%) who reported wait time was too long	N (%) who reported wait time was acceptable
Psychologist	272 (72.5)	177 (65.1)	230	100.1 (77.25)	91.3	10–365	203 (88.3)	10 (4.3)
Psychiatrist	160 (42.7)	128 (80.0)	127	127.5 (78.80)	107.0	18–341	120 (94.5)	3 (2.4)
School counsellor	105 (28.0)	12 (11.4)	69	60.9 (74.31)	21.0	8–365	48 (69.6)	16 (23.2)
Headspace	97 (25.9)	40 (41.2)	79	107.6 (89.44)	91.3	14–365	68 (86.1)	4 (5.1)
Child and Adolescent Mental Health Services	69 (18.4)	30 (43.5)	51	71.6 (65.52)	49.0	14–304	42 (82.4)	8 (15.7)
Paediatrician	50 (13.3)	37 (74.0)	33	121.9 (83.85)	101.3	14–365	28 (84.8)	4 (12.1)
Inpatient hospital stay	32 (8.5)	17 (53.1)	19	82.5 (70.14)	67.9	10–272	17 (89.5)	0 (0)
Support group	27 (7.2)	6 (22.2)	18	72.0 (78.85)	43.2	14–304	11 (61.1)	5 (27.8)
Structured psychological programme or service	25 (6.7)	9 (36.0)	15	99.1 (76.73)	91.3	14–262	9 (60.0)	3 (20.0)
Aboriginal/Torres Strait Islander medical centre	4 (1.1)	3 (75.0)	2	45.7 (21.52)	45.7	30–61	2 (100.0)	0 (0)

GPgeneral practitioner

### Comparisons with NHS benchmarks

Table 3 outlines the proportion of participants who accessed their first treatment session within the NHS benchmarks. Averaged across all primary health service providers (psychologist, headspace, psychiatrist, CAHMS), only 27.9% of participants reported a wait time of less than 6 weeks (n*=*136/487). Of these, the proportion that accessed their first treatment session within the 6-week NHS benchmark was lowest for psychiatrists (n=20/127; 15.7%), psychologists (n=67/230; 29.1%), and headspace centres (n*=*25/79; 31.6%). Over two-thirds (70.4%) had their first treatment session within 18 weeks and 29.6% waited over 18 weeks.

**Table 3:** The proportion of participants who received their first treatment session within the NHS benchmarks

	NHS	Psychologist		Psychiatrist		Headspace		Child and adolescent mental health services		All primary health services	
	%	N	%	N	%	N	%	N	%	N	%
Within 6 weeks	75	67	29.1	20	15.7	25	31.6	24	47.1	136	27.9
Within 18 weeks	95	167	72.6	77	57.2	56	70.9	43	84.3	343	70.4
>18 weeks	5	63	27.4	50	39.4	23	29.1	8	15.7	144	29.6

Note. FTwenty-four outliers were excluded.

NHSNational Health Service

### Psychological distress and perceived changes in mental health during the wait time

Across the whole sample, the mean psychological distress score was 19.40 (SD: 3.42, range: 5–25), representing a high level of distress at the time of the survey. Overall, 350 (93.3%) participants reported a distress score of 14 or above, indicating that they were experiencing clinically meaningful levels of psychological distress. Over two-thirds (67.5%, n=243/360) perceived that their feelings of sadness had worsened during their wait time, and 71.5% (n=256/363) perceived that their feelings of worry had worsened. In contrast, 13.9% (n=50/360) perceived that their feelings of sadness had reduced during their wait time and 14.6% (n=53/363) perceived that their worry had reduced.

### Associations between wait times and psychological distress among those currently waiting for their first treatment session

Participants who were currently waiting for their first treatment session reported a mean psychological distress score of 19.13 (SD: 3.83, n*=*164) with 90.2% experiencing clinically meaningful levels of psychological distress. In this group, there was a small positive correlation between psychological distress and overall wait times for all services combined (n=131, r=0.29, p=0.001). There was also a small positive correlation between psychological distress and the wait time for psychologists (r=0.35, n=93, p=0.001) and psychiatrists (r=0.30, n=43, p=0.050), such that longer wait times were associated with increased psychological distress. No other significant associations were found (p=0.101–0.983). Results using Pearson correlations were comparable in magnitude and statistical significance.

### Support from healthcare providers during the wait time

The majority of participants reported that it was ‘very’ or ‘extremely’ important (n*=*274; 73.1%) that their healthcare providers offered them support while they waited for their first treatment session. However, nearly 40% reported that they were ‘not at all’ (n*=*142; 37.9%), or only ‘slightly’ supported (n*=*131; 34.9%) during this time. When asked to select what support they had received, 38.1% (n=143) were contacted by their waitlisted provider, 31.2% (n=117) had a follow-up session with their GP, 30.9% (n=116) were given information on support services, 22.1% (n=83) were provided mental health information/brochures and 21.2% (n=79) had received a follow-up phone call from their doctor/GP.

When asked what treatment providers could have done to better support them (free response), the key themes were: increased contact from the waitlisted service (n=64/142; 45.1%, for example, ‘more check ins’, ‘greater communication’ and ‘transparency’), practical information (n=48/142; 33.8%, for example, ‘mental health strategies and resources’ and ‘online resources’) and other (n=30/142; 21.1%, for example, ‘crisis support’, ‘emotional support and validation’, ‘alternate referrals’, ‘medication’). When asked what would have helped them the most during the wait time (free response), participants (n=71/340; 20.9%) reported ‘more frequent check-ins’ and ‘greater contact from healthcare providers with updates about the status of appointment’. Participants also requested ‘resources’ (n=57/340; 16.8%), ‘emotional support’ or ‘someone to talk to’ (n=52/340; 15.3%), ‘alternate services’ or ‘referral to another mental health professional’ (n=49/340; 14.4%), ‘shorter wait times’ (n=36/340; 10.6%) and support from informal sources such as ‘parents, friends, and support groups’ (n=35/340; 10.3%).

### Sources of personal support during the wait time

Table 4 outlines the sources of support participants used and associated helpfulness ratings. Most participants turned to friends (n=338, 90.1%), parents (n=331, 88.3%) and their GP (n=305, 81.3%) for support during the wait time. Over half of the sample had used a digital source of support including web-based tools, mental health websites, helplines and mobile apps. On average, friends were rated as ‘moderately helpful’ sources of support, with all other informal, professional and digital sources mostly rated as ‘somewhat helpful’. Most participants endorsed that it was ‘very’ to ‘extremely’ important that their parents/guardians be provided with additional support to help them cope during the wait time (n*=*225/375, 60.0%), with very few reporting that it was ‘not at all’ important (n*=*23/375, 6.1%).

**Table 4:** Sources of support used by participants during the wait time (n=375)

Source of support	Used this source	Helpfulness rating	
	n (%)	M	SD
Informal sources			
Friends	338 (90.1)	3.09	1.18
Parent	331 (88.3)	2.30	1.18
Siblings	260 (69.3)	2.00	1.13
Other relative/family	225 (60.0)	1.97	1.20
Other adult	201 (53.6)	2.16	1.15
Professional sources			
GP/local doctor	305 (81.3)	2.23	1.10
School counsellor	278 (74.1)	2.17	1.22
Teacher	257 (73.3)	2.06	1.13
Year advisor or equivalent	233 (62.1)	1.94	1.15
Other MH professionals	232 (61.9)	2.35	1.21
Digital sources			
Web-based assessment tools	274 (73.3)	2.56	1.18
Mental health websites	270 (72.0)	2.40	1.21
Telephone helpline	230 (61.3)	1.93	1.17
Mental health mobile app	214 (57.1)	2.00	1.00
Online mental health programme	196 (52.2)	2.06	1.10
Online mental health chat services	189 (50.4)	2.10	1.10
Online mental health support forums	165 (44.0)	2.25	1.31

Note. Percentages are reported for the subset of participants that selected each source of support. The range for each source of support listed is 1 (not at all helpful) to 5 (extremely helpful).

GPgeneral practitionerMHmental health

### Coping behaviours used during the wait time

As outlined in table 5, 92.8% (n=348) of participants used one or more maladaptive coping behaviours during the wait time such as spending more time alone (n=270; 72.0%) and sleeping (n=260; 69.3%). A total of 87.5% (n=328) used one or more help-seeking behaviours such as searching the internet to find mental health information (n=240; 64.0%) and reaching out to friends via SMS (n=199, 53.1%). Over two-thirds reported that they had engaged in one or more risky coping behaviours (n=284, 75.7%) such as self-harm (n=209; 55.7%) and skipping school (n=174; 46.4%).

**Table 5:** Coping behaviours used by participants during the wait time (n=375)

	N	%
Maladaptive behaviours	348	92.8
Spending more time by myself	270	72.0
Spending more time sleeping	260	69.3
Spending more time on social media	244	65.1
Spending more time at home	244	65.1
Eating more treat food and/or takeaway food	176	46.9
Spending more time online gaming	106	28.3
Help-seeking behaviours	328	87.5
Searching the internet for information about mental health	240	64.0
Speaking with friends over text message	199	53.1
Seeking support from friends	166	44.3
Speaking with a school counsellor, teacher or other school support	120	32.0
Speaking with friends over a phone call	111	29.6
Risky behaviours	284	75.7
Self-harming	209	55.7
Skipping school	174	46.4
Drinking alcohol	102	27.2
Vaping	86	22.9
Using cannabis	66	17.6
Smoking cigarettes	49	13.1
Using other drugs	40	10.7
Adaptive behaviours	272	72.5
Writing down how I feel (eg, journaling)	116	30.9
Doing more exercise or sport	112	29.9
Doing activities that help me relax	111	29.6
Reading books	100	26.7
Doing more activities I enjoy	98	26.1
Taking up a new activity, sport or hobby	90	24.0
Meeting up with friends or becoming more social	88	23.5
Improving or changing my diet	87	23.2

Note. Total n and % for each category were calculated based on whether participants endorsed at least one strategy in that category.

### Self-reported attendance at the first treatment session

Among those who were currently waiting, 78.7% (n*=*129/164) reported that they were likely to attend their first treatment session and 14.7% (n*=*24/164) reported that they were unlikely to attend. The most common reasons for likely non-attendance were ‘the wait time was too long’ (n*=*13/24; 54.2%), ‘don’t want to go’ (n*=*13/24; 54.2%), and ‘couldn’t be bothered’ (n*=*11/24; 45.8%). Four participants in this subgroup (n*=*4/24; 16.6%) selected the response ‘I don’t need it anymore, I feel better’. Among those who had previously waited, almost all reported that they attended their first session (n*=*203/211; 96.2%); however, ‘the wait time was too long’ (n*=*6/8; 75%) and ‘didn’t want to go’ (n*=*3/8; 37.5%) were the main reasons for self-reported non-attendance in this subgroup.

## Discussion

### Primary findings

This study presents a cross-sectional examination of adolescents’ experiences of wait times for mental health treatment for anxiety and depression in Australia. Consistent with the hypotheses, the average self-reported wait times for several mental health treatment providers exceeded 100 days. Most adolescents in this sample were waiting to access psychologists, psychiatrists and headspace centres for more than 3 months and the majority felt that their wait times were ‘too long’. While there was significant variation in wait times across services and between participants, these did not differ between states. Wait times for psychiatrists were significantly longer in metropolitan locations, whereas wait times for paediatricians and school counsellors were longer in regional areas. The average self-reported wait times found in this study were more than three times higher than previous Australian reports,^3^ although consistent with more recent data on wait times for psychologists.^12^ Overall, these results indicate significant gaps between adolescents’ need for mental health treatment for anxiety and depression and its timely availability in Australia.

In further support of our hypotheses, longer wait times were associated with higher levels of psychological distress, and over two-thirds of participants felt their mental health had worsened during the wait time. Moreover, many of the maladaptive and risky coping behaviours used by participants may have signified further deterioration of symptoms (eg, sleeping, social withdrawal, self-harm). While some participants felt their mental health had improved during the wait time, our results are consistent with several past studies that observed declines in mental health among young people waiting for care.^36^,^39^ However, as this study is cross-sectional, there was no evidence to suggest that wait times caused poorer mental health in young people. Rather, the results may reflect the natural illness progression of anxiety and depression among this sample and their greater need for treatment. Regardless, the findings suggest that the wait time for mental health treatment is likely to be a period of significant vulnerability for many adolescents, characterised by high levels of psychological distress, perceived worsening of mental health and engagement in maladaptive and risky coping behaviours.

### Implications for clinical practice

This study confirms that many adolescents were provided with nil to minimal support from their healthcare providers during the wait time, despite the majority feeling that it was important. Interestingly, the support preferences of adolescents were low intensive, non-clinical and communication-based. Specifically, adolescents requested more contact and ‘check-ins’ from their waitlisted service provider, which could be administered by practice staff or automated through technological platforms such as SMS. A digital system that periodically contacts adolescents with updates about their upcoming appointments and provides relevant web-based tools and positive coping strategies may be beneficial to adolescents during the wait time given their prior positive experiences with digital resources. Service designers should actively engage with adolescent treatment seekers to further explore and co-design such an approach. Moreover, the high referral rates and interim care provided by GPs further confirm the importance of their role in mental health service provision in Australia. Future research would benefit from examining GPs’ understanding of wait times, the impacts on their treating behaviour and how to best support GPs in providing interim care to their youth patients on wait lists for mental health treatment.

In this study, most participants reported attending their first treatment session or were likely to, despite experiencing long wait times. This finding contrasts with several studies that imply longer wait times lead to treatment disengagement across adolescents.^13^,^16^ Our results may reflect the ‘sunken cost’ associated with longer wait times, such that the time, effort and resources involved in accessing scarce treatment led to higher retention levels in youth. This finding may also reflect the higher levels of motivation and commitment to treatment among this sample, which may or may not be due to longer wait times. As most participants were in secondary school, their treatment adherence may have also been sustained through parental, familial and school support. As such, different patterns of service use may be found in other samples and studies with longer periods of observation. However, long wait times were reported as the primary reason that non-attenders did not start their treatment. This suggests that long wait times may reduce treatment uptake in a subgroup of adolescent help-seekers, and future research may benefit from examining this pattern of treatment engagement in more detail. Moreover, international studies have found that many parents facing long wait times place their adolescent children on multiple wait lists, which may further exacerbate wait times.^40 41^ Future studies may benefit from examining whether long wait times lead parents and adolescents to place themselves on multiple waitlists for the same type of treatment provider, inadvertently contributing to longer wait times and increased demand for some providers in Australia.

### The call for national standards

The overall wait times reported in this study exceeded the NHS standards, with only one in four young people reporting a wait time of less than 6 weeks and one-third waiting longer than 18 weeks. Given that the introduction of transparent wait time standards in the UK and other countries has reduced wait times significantly,^19 22^ our results support the call for transparent wait time monitoring and reporting for mental health treatment in Australia. This approach may improve the timely provision of mental health treatment to both adolescents and adults. As a start, this could be achieved through mandatory reporting from any mental health professional that benefits from the Better Access initiative—a federal government programme that provides subsidised mental healthcare to Australian residents.^42^ This approach would also enable the identification of locations and treatment services with greater need as well as the objective data needed to evaluate the impact of systemic changes on wait time durations.^43^ Future research should use evidence-based approaches that involve service users, including clinicians, parents and families, schools and young people to determine acceptable wait time targets for the Australian context.^44^

### Limitations

This study is an important step in understanding the wait times for mental health treatment for anxiety and depression in Australia in the absence of robust national data. This study is strengthened by the involvement of adolescents with lived experience in the survey design and recruitment methods. This study is also strengthened by the representation of adolescents from hard-to-reach groups, including those who identify as gender and/or sexuality diverse. The diversity rates reported were similar to other mental health trials of adolescents in Australia^45^,^47^ but were somewhat higher than the general population.^48^ As the study did not specifically target these groups in recruitment, these rates may reflect the increased need for mental health treatment among these youth and/or their higher levels of help-seeking.^49 50^ These rates may also reflect the allyship of the Black Dog Institute for gender and/or sexuality-diverse adolescents in Australia. Nevertheless, the high proportion of LGBTQIA+ respondents may limit the generalisability of these findings to other demographic groups.

Due to the sampling method, the study does not represent the experiences of adolescents who accessed their first treatment session within a short time frame (eg, less than 1 week) or those satisfied with their wait time. Additionally, the definition of ‘first appointment’ did not distinguish a psychotherapy session from other types of first appointments such as intake assessments, given that adolescents who were waiting for care could not be expected to know this distinction. Therefore, the wait times for the services that may use intake assessments, such as headspace or CAMHS, may be an underestimation of the length of time taken to receive psychological therapy. The use of self-report data may also be limited by poor or inaccurate recall. Different results may be found in treatment provider records or when more objective measures are used. Seasonal variations in wait times reported by other service providers^3^ were also not captured by this study due to the time-limited and cross-sectional study design. As such, different wait times may be found when data is collected over longer periods. Finally, the current study did not measure the presence of co-occurring complexities that may have inflated wait times, such as the need for specialised mental healthcare (eg, trauma, eating disorders, neurodivergence). Future work may benefit from greater attempts to understand how treatment-seeking may be influenced by symptom severity, comorbidities or additional psychosocial needs.

### Conclusion

This study is the first to examine Australian adolescents’ wait times for the treatment of anxiety and depression. Findings indicated that many Australian youth face extended delays across several treatment providers, with many adolescents perceiving the wait times as too long. The findings highlight the need for national transparency and benchmarking of wait times for mental health treatment providers in Australia. Many participants felt unsupported by their referred providers and that their mental health had worsened during the wait time, with many engaging in unhelpful coping behaviours. As such, more research is needed to determine best practices for addressing young people’s mental health needs while they await professional treatment for anxiety and depression, with adolescent perspectives informing these practices to ensure their relevance and effectiveness.

## supplementary material

10.1136/bmjopen-2024-087342 online supplemental file 1

## Data Availability

All data relevant to the study are included in the article or uploaded as supplementary information.

## References

[1] Lawrence D, Hafekost J, Johnson SE (2016). Key findings from the second Australian Child and Adolescent Survey of Mental Health and Wellbeing. Aust N Z J Psychiatry.

[2] World Health Organisation (2021). Mental health of adolescents.

[3] National Youth Mental Health Foundation (2019). Increasing demand in youth mental health: A rising tide of need. https://headspace.org.au/assets/Uploads/Increasing-demand-in-youth-mentalh-a-rising-tide-of-need.pdf.

[4] Kowalewski K, McLennan JD, McGrath PJ (2011). A preliminary investigation of wait times for child and adolescent mental health services in Canada. J Can Acad Child Adolesc Psychiatry.

[5] Smith J, Kyle RG, Daniel B (2018). Patterns of referral and waiting times for specialist Child and Adolescent Mental Health Services. Child Adolesc Ment Health.

[6] Public Health Scotland (2013). Child and Adolescent Mental Health Services waiting times in Scotland. Quarter ending. https://publichealthscotland.scot/media/8975/2021-09-07-camhs-waiting-times-report.pdf.

[7] Stringer H (2023). Providers predict longer wait times for mental health services. Here’s who it impacts most: Psychologists worry that patients from marginalized populations, particularly people of color, will suffer most amid a worsening workforce shortage. Monitor Psychol.

[8] Edbrooke-Childs J, Deighton J (2020). Problem severity and waiting times for young people accessing mental health services. BJPsych Open.

[9] Lewis AK, Harding KE, Snowdon DA (2018). Reducing wait time from referral to first visit for community outpatient services may contribute to better health outcomes: a systematic review. BMC Health Serv Res.

[10] Australian Government Productivity Commission (2022). Report on Government Services 2022: 13 Services for mental health. Impact of COVID-19 on data for the Services for mental health section.

[11] Mulraney M, Lee C, Freed G (2021). How long and how much? Wait times and costs for initial private child mental health appointments. J Paediatr Child Health.

[12] Australian Psychological Society (2021). Balancing caseloads and surging demand: Your experience and what we are doing. InPsych.

[13] Westin AML, Barksdale CL, Stephan SH (2014). The effect of waiting time on youth engagement to evidence based treatments. Community Ment Health J.

[14] Gallucci G, Swartz W, Hackerman F (2005). Impact of the wait for an initial appointment on the rate of kept appointments at a mental health center. *Psychiatr Serv*.

[15] Sherman ML, Barnum DD, Buhman-Wiggs A (2009). Clinical intake of child and adolescent consumers in a rural community mental health center: does wait-time predict attendance?. Community Ment Health J.

[16] Williams ME, Latta J, Conversano P (2008). Eliminating the wait for mental health services. J Behav Health Serv Res.

[17] Black G, Roberts RM, Li-Leng T (2012). Depression in rural adolescents: relationships with gender and availability of mental health services. Rural Remote Health.

[18] Punton G, Dodd AL, McNeill A (2022). “You’re on the waiting list”: An interpretive phenomenological analysis of young adults’ experiences of waiting lists within mental health services in the UK. PLoS One.

[19] OECD Publishing (2021). A New Benchmark for Mental Health Systems: Tackling the Social and Economic Costs of Mental Ill-Health, OECD Health Policy Studies, paris.

[20] Department of Health and Social Care (2014). Department of Health and Social Care. Achieving Better Access to Mental Health Services by 2020.

[21] NHS England (2015). Improving Access to Psychological Therapies (IAPT) aiting Times Guidance and FAQ’s.

[22] NHS 75 Digital (2019). Psychological Therapies: reports on the use of IAPT services, England April 2019 final including reports on the IAPT pilots.

[23] Potter C (2022). NHSE to begin ‘implementation plan’ for new mental health waiting time standards. Pulse (Basel).

[24] Yang F, Wangen KR, Victor M (2022). Referral assessment and patient waiting time decisions in specialized mental healthcare: an exploratory study of early routine collection of PROM (LOVePROM). BMC Health Serv Res.

[25] Kelly AB, Halford WK (2007). Responses to ethical challenges in conducting research with Australian adolescents. Aust J Psychol.

[26] Australian Bureau of Statistics (ABS) (2016). 1270.0.55.001 - Australian Statistical Geography Standard (ASGS): Volume 1 - Main Structure and Greater Capital City Statistical Areas.

[27] Batterham PJ, Sunderland M, Carragher N (2016). The Distress Questionnaire-5: Population screener for psychological distress was more accurate than the K6/K10. J Clin Epidemiol.

[28] Batterham PJ, Sunderland M, Slade T (2018). Assessing distress in the community: psychometric properties and crosswalk comparison of eight measures of psychological distress. Psychol Med.

[29] Werner-Seidler A, Huckvale K, Larsen ME (2020). A trial protocol for the effectiveness of digital interventions for preventing depression in adolescents: The Future Proofing Study. Trials.

[30] IBM Corp (2021). SPSS Version 28.

[31] Cobanoglu C, Cavusoglu M, Turktarhan G (2021). A beginner’s guide and best practices for using crowdsourcing platforms for survey research: The Case of Amazon Mechanical Turk (MTurk). *JGBI*.

[32] Clarke V, Braun V (2013). Teaching thematic analysis: Overcoming challenges and developing strategies for effective learning. Psychologist.

[33] Tuckett AG (2005). Applying thematic analysis theory to practice: a researcher’s experience. Contemp Nurse.

[34] Bengtsson M (2016). How to plan and perform a qualitative study using content analysis. *NursingPlus Open*.

[35] Erlingsson C, Brysiewicz P (2017). A hands-on guide to doing content analysis. Afr J Emerg Med.

[36] Aisbett DL, Boyd CP, Francis KJ (2007). Understanding barriers to mental health service utilization for adolescents in rural Australia. Rural Remote Health.

[37] Iskra W, Deane FP, Wahlin T (2018). Parental perceptions of barriers to mental health services for young people. Early Interv Psychiatry.

[38] Leijdesdorff S, Klaassen R, Wairata D-J (2021). Barriers and facilitators on the pathway to mental health care among 12-25 year olds. *Int J Qual Stud Health Well-being*.

[39] Reichert A, Jacobs R (2018). The impact of waiting time on patient outcomes: Evidence from early intervention in psychosis services in England. Health Econ.

[40] Reid GJ, Cunningham CE, Tobon JI (2011). Help-seeking for children with mental health problems: parents’ efforts and experiences. Adm Policy Ment Health.

[41] Shanley DC, Reid GJ, Evans B (2008). How parents seek help for children with mental health problems. Adm Policy Ment Health.

[42] Department of Health and Aged Care, Australian Government (2023). Better access to psychiatrists, psychologists and general practitioners through the MBS (Better Access) initiative.

[43] Rastpour A, McGregor C (2022). Predicting Patient Wait Times by Using Highly Deidentified Data in Mental Health Care: Enhanced Machine Learning Approach. JMIR Ment Health.

[44] Eichstedt JA, Singh D, Chen S (2021). Who Should Be Seen When? Establishing Wait Time Benchmarks for Children’s Mental Health. Canadian J Comm Mental Health.

[45] O’Dea B, Li SH, Subotic-Kerry M (2024). The MobiliseMe study: A randomised controlled efficacy trial of a cognitive behavioural therapy smartphone application (ClearlyMe®) for reducing depressive symptoms in adolescents.

[46] O’Dea B, Han J, Batterham PJ (2020). A randomised controlled trial of a relationship-focussed mobile phone application for improving adolescents’ mental health. *J Child Psychol Psychiatry*.

[47] Shvetcov A, Whitton A, Kasturi S (2023). Machine learning identifies a COVID-19-specific phenotype in university students using a mental health app. Internet Interv.

[48] Werner-Seidler A, Maston K, Calear AL (2023). The Future Proofing Study: Design, methods and baseline characteristics of a prospective cohort study of the mental health of Australian adolescents. Int J Methods Psychiatr Res.

[49] Shorey S, Ng ED, Wong CHJ (2022). Global prevalence of depression and elevated depressive symptoms among adolescents: A systematic review and meta-analysis. Br J Clin Psychol.

[50] Wilson C, Cariola LA (2020). LGBTQI+ Youth and Mental Health: A Systematic Review of Qualitative Research. Adolescent Res Rev.

