# Supplementary material for: While they wait: a cross-sectional survey on wait times for mental health treatment for anxiety and depression for adolescents in Australia
Source: BMJ Open. 2025 Mar 24;15(3):e087342. doi: 10.1136/bmjopen-2024-087342 (PMC11934408; doi:10.1136/bmjopen-2024-087342)
Supplement: Supplementary file 2 [file bmjopen-2024-087342.reviewer_comments.pdf]

PEER REVIEW HISTORY

BMJ Open publishes all reviews undertaken for accepted manuscripts. Reviewers are asked to complete a checklist review form and are provided with free text boxes to elaborate on their assessment. These free text comments are reproduced below.

ARTICLE DETAILS

Title (Provisional)

While they wait: A cross-sectional survey on wait times for mental health treatment for anxiety and depression for adolescents in Australia

Authors

Subotic-Kerry, Mirjana; Borchard, Thomas; Parker, Belinda; Li, Sophie H; Choi, Jayden; Long, Emma V; Batterham, Philip J; Whitton, Alexis; Gockiert, Aniela; Spencer, Lucinda; O'Dea, Bridianne

---

VERSION 1 - REVIEW

**Reviewer**	**1**
**Name**	**Adams, Danielle R.**
**Affiliation**	**Washington University in St Louis**
**Date**	**27-Jun-2024**
**COI**	**I have no competing interests to disclose.**

This study presents a cross-sectional examination of Australian adolescents'

experiences of wait times for mental health treatment for anxiety and depression. Results shed light into wait times and adolescents experiences of these and provide important policy implications.

Introduction:

• You define wait times as the time between initial contact and first appointment – is first appointment defined as intake, or the first psychotherapy appointment? In the U.S., folks may be seen faster for intake and then have another long wait between intake and first psychotherapy appointment. How do you think about this distinction in Australia and your study?

• Introduction is very well written and sets up the study well, nice job.

Methods:

• Please clarify where you are discussing when you say "population size of N=97, 500" – where in Australia?

• Recruitment – please say more about what the "Black Dog Institute" is for unfamiliar readers; why should readers trust this as a recruitment source for this study?

• Please provide more information about the “4-item Gillick Competence Test” what is it? Provide a sample question and more information about why it was selected

• “Participants were asked whether they had ever been formally diagnosed with depression and/or anxiety by a health professional and whether they were currently taking medication prescribed by a health professional for depression and/or anxiety.” – please provide more information on the questions you used to assess depression and anxiety. Were they validated and reliable measures? If not, why did you not use validated and reliable measures when they are widely available? Please state this as a limitation if you did not use validated and reliable measures; self-report of depression and anxiety has limitations.

• Regarding the “11 mental health treatment providers” please list the options. Additionally, how do you handle this issue of folks, especially adolescents, not reporting accurately on what type of provider they were seeking? That is, an adolescent may not know whether they are seeking mental health treatment from a psychologist or social worker, they just know they are seeking mental health treatment – please discuss.

• Please provide examples of the 17 sources of “personal support” that adolescents could select in that section of the methods.

• Similarly, please provide examples of the coping behaviors adoelscents could have selected from, at least one for each ‘maladaptive, risky, help-seeking, healthy”

• Please provide examples of the 11 “reasons for non-attendance” offered to adolescents. Was a write in offered to adolescents here?

Results

• Please provide more information on why only 375/780 participants were eligible for the study.

• “Participants utilised an average of 2.29 (SD: 1.31, range: 1-9) treatment providers” – is utilized the right word here? Or perhaps “reached out to / scheduled with”?

• Please define “GP” upon first use

• “the average wait times for the most common treatment providers all exceeded three months.” – please list the actual wait time average and median instead of just exceeded three months. Also state the name of the most common treatment provider

• How did wait time vary by provider type?

• “Across all treatment providers, most participants perceived that their wait time was “too long”. How many is most?

• Please contextualize “mean psychological distress score of 19.13” – is that high or low?

• You mention identifying “key themes” from the free response questions – please include a new section in the methods on how you coded these free response questions to identify these key themes.

Discussion

• Please define 'SMS'

• Please clarify this sentence, it is confusing as stated "As young people endorsed the helpfulness of some digital resources, a system that contacts young people periodically about their appointment, provides links to web-based tools and information, as well as positive coping behaviours, is likely to be regarded as helpful to adolescents on wait lists for anxiety and depression treatment"

• Please clarify what you mean by "over servicing" of treatment providers

• Section on "call for national standards" is fantastic, I appreciate the policy relevance

• Limitations – High rates of LGBTQ+ respondents is also a strength!

• I see another strength of your study as including youth in the design of the survey and recruitment; I would consider writing about this as a strength in your discussion and in your conclusion talk about how youth should be involved in creating those best practices for addressing young peoples mental health needs

---

**Reviewer**	**2**
**Name**	**Mulraney, Melissa**
**Affiliation**	**Murdoch Childrens Research Institute**
**Date**	**08-Jul-2024**
**COI**	**Nil**

Thank you for the opportunity to review this well written article on a topic of great importance. Overall, the manuscript is very well written and presents a clear description of the study and findings. I have one major concern and two minor issues that need to be addressed before I can endorse the manuscript for publication.

Most importantly, although the methods described to detect and remove fraudulent responses are comprehensive, the pattern of some of the data makes me question the validity of some of the included responses. The authors provide in the discussion some explanation for why the rates of LGBTQIA+ are so much higher than expected, this plus the high rates of non-binary and 'another identity' genders, and ATSI respondents make one question the data particularly as these groups were not targeted in recruitment. Also, the range of wait times for school counsellor goes up to 727 days. Given this service, this does not seem a realistic time frame. Given this, perhaps the authors could conduct a sensitivity analysis whereby the analyses are repeated only for those participants who have provided responses to the open-ended questions that clearly indicate an authentic responder. If a similar pattern of results is found this would provide much more confidence in the study as a whole.

Two minor issues:

- The inclusion/exclusion criteria are a bit confusing. It seems that exclusion "(i) currently waiting for a follow-up treatment session with a mental health professional or service that they had accessed previously" contradicts the inclusion criteria of "or had previously waited (in the last 12 months)

longer than one week to access their first session of mental health treatment with a mental health professional or service for symptoms of anxiety and/or depression”.

- History of mental health is limited to anxiety/depression, this could be a limitation as many young people with these conditions have a history of other mental health and neurodevelopmental conditions which would influence their familiarity with accessing the mental health system and could impact on the findings.

VERSION 1 - AUTHOR RESPONSE

Reviewer 1

1. **You define wait times as the time between initial contact and first appointment – is first appointment defined as intake, or the first psychotherapy appointment? In the U.S., folks may be seen faster for intake and then have another long wait between intake and first psychotherapy appointment. How do you think about this distinction in Australia and your study?**

RESPONSE: We agree that our attempt to define wait times in the introduction may be misplaced given differences in international contexts. We have removed this definition from the Introduction and included a comment in the Limitations section.

2. **Please clarify where you are discussing when you say “population size of N=97, 500” – where in Australia?**

RESPONSE: Thank you for your helpful comment regarding the population size. The “population size of N=97,500” refers to the estimated number of Australian adolescents aged 13-17 years who meet criteria for a clinical diagnosis of anxiety and/or depression and are likely to seek mental health treatment. This figure was derived from national mental health data sources, specifically a large national survey, the Australian Child and Adolescent Survey of Mental Health and Wellbeing, to represent the target population relevant to our study.

We have updated the manuscript to specify this context and clarify that this is a national estimate of Australian adolescents with mental health needs related to anxiety and/or depression. The sample size section in the methods now reads:

“The population size reflects the estimated number of adolescents in Australia aged 13-17 years who meet the criteria for a clinical diagnosis of anxiety and/or depression and are likely to seek mental health treatment based on a nationally representative sample[1].”

3. **Recruitment – please say more about what the “Black Dog Institute” is for unfamiliar readers; why should readers trust this as a recruitment source for this study?**

RESPONSE: Thank you for this suggestion to better communicate the credibility of our recruitment methods. The Black Dog Institute was the sponsor of this research. The Institute is a leading Australian mental health research institute affiliated with the University of New South Wales. It is internationally recognised for its evidence-based research and development of mental health interventions, particularly in the areas of mood disorders, suicide prevention, and digital mental

health. The Institute collaborates with healthcare providers, community organisations, and academic institutions to enhance mental health literacy, promote early intervention, and improve access to care.

Recruiting through the Black Dog Institute offered a trusted and reputable avenue to reach our target population and ensured that the study maintained high ethical and scientific standards. We have expanded the manuscript to include this background information, providing additional context for readers unfamiliar with the Black Dog Institute. This section now reads:

"Participants were recruited via paid social media campaigns on Facebook, Twitter, Instagram, and LinkedIn (for parents and carers to promote to youth). Study information was also published on the research sponsor's (Black Dog Institute) website and circulated through their clinical service partners. The Black Dog Institute is a mental health research institute in Australia affiliated with the University of New South Wales. The Institute's website promotes research participation opportunities to a range of diverse audiences. All recruitment materials were submitted and approved by UNSW HREC."

4. **Please provide more information about the "4-item Gillick Competence Test" what is it? Provide a sample question and more information about why it was selected.**

RESPONSE: The Gillick Competence Test has been used by the authors in several studies to assess the capacity of a person under the age of 18 to consent to research participation without active parental consent. The test is based on the legal framework established in the UK case *Gillick v West Norfolk and Wisbech Area Health Authority* which allows minors to make independent decisions if they demonstrate sufficient understanding and intelligence to appreciate the nature, purpose, and potential consequences of their participation. A Gillick Competence Test is an attempt to ensure ethical research practice by assessing a young person's ability to provide informed consent autonomously, which is particularly important in studies involving potentially sensitive mental health topics.

In this study, the 4-item Gillick Competence Test included questions designed to evaluate the participant's comprehension of what the study involved, who the research was being conducted by, the voluntary nature of participation, and their understanding of who their survey data would be shared with. This approach helped ensure that participants had a clear understanding of the study before providing their consent.

We have added more detail in the manuscript regarding the purpose and structure of the test, along with examples of the questions, to enhance clarity for readers:

"Before commencing the survey, participants were presented with the Participant Information sheet and were required to pass screening questions and a 4-item Gillick Competence Test[25]. This test was used to measure the capacity of adolescents aged under 18 years to provide informed consent to participate in research. Four questions, answered using three multiple choice options, tested the participant's comprehension of what the research study involved ("This research study involves…"), who the research study was being conducted by ("This research is being conducted by…"), the voluntary nature of participation ("Do I have to finish the survey?") and who their responses would be shared with ("Your responses to this survey will be shared with…"). Individuals who did not complete the items correctly were excluded. For a full copy, please see Appendix A.

5. **Participants were asked whether they had ever been formally diagnosed with depression and/or anxiety by a health professional and whether they were currently taking**

medication prescribed by a health professional for depression and/or anxiety." – please provide more information on the questions you used to assess depression and anxiety. Were they validated and reliable measures? If not, why did you not use validated and reliable measures when they are widely available? Please state this as a limitation if you did not use validated and reliable measures; self-report of depression and anxiety has limitations.

<u>RESPONSE:</u> The current study was focussed on aspects of the help-seeking journey for anxiety and depression and therefore targeted young people who had sought help for these symptoms in the past 12 months. The current study was not designed to validate their depressive or anxiety symptoms or confirm their diagnosis or suitability for treatment. Participants were asked two self-report questions about their mental health history: (1) "Have you ever been formally diagnosed with depression and/or anxiety by a health professional?" [to indicate professional help-seeking and formal diagnosis] and (2) "Are you currently taking medication prescribed by a health professional for depression and/or anxiety?" [to indicate use of prescription medications, and potential illness severity] to confirm participants' treatment-seeking status rather than to measure current symptomatology or severity of depression and anxiety. In addition, because of the help-seeking period we were interested in exploring (i.e., young people who had waited for services between a week and up to 12 months, measuring the status of their depressive and anxiety symptoms at screening to validate their treatment-seeking status at screening would have been irrelevant.

We included a validated self-report scale of psychological distress to measure participants' current state of distress at the time of the survey. We recognise that validated and reliable measures, such as the Patient Health Questionnaire – 9 (PHQ-9) or the Generalised Anxiety Disorder Questionnaire – 7 (GAD-7), are available for assessing current symptoms of depression and anxiety in young people. However, due to the study's primary focus on young people's experience of wait times for mental health treatment rather than clinical diagnosis, we opted for these brief self-report questions. In addition, young people with Lived Experience were involved in designing and reviewing this survey and they did not see it as valuable or suitable to have their depressive/anxiety symptoms re-measured by this survey.

6. **Regarding the "11 mental health treatment providers" please list the options. Additionally, how do you handle this issue of folks, especially adolescents, not reporting accurately on what type of provider they were seeking? That is, an adolescent may not know whether they are seeking mental health treatment from a psychologist or social worker, they just know they are seeking mental health treatment – please discuss.**

<u>RESPONSE:</u> The treatment provider options included general practitioners (GPs), psychologists, psychiatrists, social workers, counsellors, school counsellors, mental health nurses, occupational therapists specialising in mental health, peer support workers, helpline services, and online/digital mental health programs. These options were selected to represent the range of providers that adolescents in Australia commonly access for mental health support.

Our survey design recognises that adolescents may not always know the specific qualifications or titles of the professionals they are referred to or seeking help from. To address this, we included a free response "other" option to capture instances where participants were uncertain about their provider type. We have revised the method section in the manuscript to reflect these options.

7. **Please provide examples of the 17 sources of "personal support" that adolescents could select in that section of the methods.**

RESPONSE: We have updated the relevant section of the methods to include the 17 options that adolescents could select, including family members (e.g., parents, siblings), friends, school staff, coaches, religious or spiritual leaders, online support groups, and mental health apps. Please see the changes highlighted in yellow in the attached manuscript. Please note that a full copy of the survey and the response options is provided in Appendix A.

8. **Similarly, please provide examples of the coping behaviors adolescents could have selected from, at least one for each 'maladaptive, risky, help-seeking, healthy"**

RESPONSE: We have revised the methods section to include an example of the coping behaviour for each category to clarify our approach. This section now reads:

"For analysis, each behaviour was collapsed into one of four categories: maladaptive (e.g., spending more time online gaming), risky (e.g., self-harming), help-seeking (e.g., seeking support from friends), adaptive (e.g., doing more exercise or sport)."

9. **Please provide examples of the 11 "reasons for non-attendance" offered to adolescents. Was a write in offered to adolescents here?**

RESPONSE: In the methods section, we have added examples of the 11 reasons listed in the survey which included a free response or "write in" option for adolescents:

"Reasons for non-attendance included, I don't need it anymore because I feel better, I found an earlier session somewhere else, I have had to wait for too long, I can't be bothered, I might forget, I don't have the money, I don't want to go, The session is too far away from me, I don't have any transport to get there, I feel too worried and/or sad to go, I am unsatisfied with the service, A different reason (please tell us in the text box)."

10. **Please provide more information on why only 375/780 participants were eligible for the study.**

RESPONSE: We have provided clarification in the manuscript to ensure transparency in our sample selection process. The Data analyses and Results have now been updated to read:

"Researchers reviewed suspected fraudulent responses, and discrepancies were resolved by a third rater (see Supplementary Material for additional information). Fraudulent and duplicate responses were detected by comparing participants' details (email, postcode, IP addresses) and response patterns across the survey. Participants who completed the survey faster than 40% of the average completion time for the entire sample were removed as recommended by Cobanoglu et al.[31]"

"A total of 780 respondents were assessed for study eligibility and 92 were excluded due to being ineligible to participate (*n*=40) or failing the Gillick Competence Test (*n*=52). A further 313 responses were excluded due to being judged as invalid/fraudulent (*n*=211), incomplete (*n*=82), or completed too quickly (*n*=20)."

11. **Participants utilised an average of 2.29 (SD: 1.31, range: 1-9) treatment providers" – is utilized the right word here? Or perhaps "reached out to / scheduled with"?**

RESPONSE: In this sentence, utilised is used according to its definition “to make use of”. However, upon your request we have amended this to be “initiated an appointment...”:

“Participants initiated appointments with an average of 2.29 (SD: 1.31, range: 1-9) treatment providers.”

12. **Please define “GP” upon first use**

RESPONSE: The term GP has been defined upon first use on page 12.

13. **the average wait times for the most common treatment providers all exceeded three months.” – please list the actual wait time average and median instead of just exceeded three months. Also state the name of the most common treatment provider**

RESPONSE: To avoid repetition between the Tables and manuscript text, given the strict word limits on the paper, we have not repeated the wait times for each of the 11 service providers in text. Instead, we have referenced Table 2 more explicitly to encourage readers to refer to the Tables where this information is provided.

14. **How did wait time vary by provider type?**

RESPONSE: We have revised the results to reflect more information about how wait times varied by provider type. Please see the changes in the Results section highlighted in yellow in the revised manuscript.

15. **“Across all treatment providers, most participants perceived that their wait time was “too long”. How many is most?**

RESPONSE: We have provided greater clarity and this section now reads:

“Across all treatment providers, most participants (*n*=550/655, 84%) perceived that their wait time was “too long”.”

16. **Please contextualize “mean psychological distress score of 19.13” – is that high or low?**

RESPONSE: For greater clarity and transparency, we have included the following changes to the methods section describing psychological distress as measured by the Distress Questionnaire-5:

“Total scores range from 5 to 25 with higher scores indicating greater psychological distress, and a threshold of ≥14 as the clinical cut-off.”

Additionally, we have amended the results section to read:

“Across the whole sample, the mean psychological distress score was 19.40 (*SD*: 3.42, range: 5-25), representing a high level of distress at the time of the survey. Overall, 350 (93.3%) participants reported a distress score of 14 or above, indicating that they were experiencing clinically meaningful levels of psychological distress.”

17. **You mention identifying "key themes" from the free response questions – please include a new section in the methods on how you coded these free response questions to identify these key themes.**

RESPONSE: We have revised the data analysis section to include more detail about how we coded each free response question to identify key themes. This section now reads:

"Qualitative (free response) data were analysed using Clarke and Braun's (2013)[32] six-stage thematic analysis guidelines, which allow for identifying and interpreting patterns of meaning within data[33]. Given these questions were open-ended, an inductive approach was used to develop a coding framework.[34,35] The analysis involved an iterative process of reading and coding responses and then organising codes into broader themes. Two primary coders (TB and EL) independently coded a subset of responses for each free response question to create a preliminary framework, resolving discrepancies through discussion. The revised framework for each free response question was then applied to all responses, and codes were compared for consistency. Any discrepancies were resolved by a third independent rater (MSK), ensuring consistency in code descriptions".

18. **Please define 'SMS'**

RESPONSE: The term SMS (Short Messaging Service) has been defined upon first use on page 23.

19. **Please clarify this sentence, it is confusing as stated "As young people endorsed the helpfulness of some digital resources, a system that contacts young people periodically about their appointment, provides links to web-based tools and information, as well as positive coping behaviours, is likely to be regarded as helpful to adolescents on wait lists for anxiety and depression treatment"**

RESPONSE: We have revised the sentence to better explain how digital resources and communication could be helpful to young people awaiting mental health treatment:

"A digital system that periodically contacts adolescents with updates about their upcoming appointments and provides relevant web-based tools and positive coping strategies may be beneficial to adolescents during the wait time given their prior positive experiences with digital resources."

20. **Please clarify what you mean by "over servicing" of treatment providers**

RESPONSE: By "over-servicing" of treatment providers, we refer to the potential situation where adolescents (or their parents) are placed on multiple waitlists for mental health services simultaneously due to the anticipation of long wait times. This practice can lead to inefficiencies in the system, as multiple providers may be holding a spot for the same individual, which can unnecessarily extend wait times for other adolescents seeking services. In such cases, the demand for services appears higher than it is, not because more individuals are seeking care, but because each individual is reserving multiple spots, thereby contributing to longer wait times across providers. We have revised this statement and removed the term 'over-servicing" for greater clarity and the revised section in the Discussion now reads:

"Future studies may benefit from examining whether long wait times lead parents and adolescents to place themselves on multiple waitlists for the same type of treatment provider, inadvertently contributing to longer wait times and increased demand for some providers in Australia."

21. **Section on "call for national standards" is fantastic, I appreciate the policy relevance**

RESPONSE: Thank you for your positive feedback on the section calling for national standards. We are pleased that you found the policy relevance of this aspect valuable, as we aimed to highlight the importance of establishing benchmarks and transparency in wait times for mental health services. We agree that this paper offers only an initial exploration of this issue and more work to quantify wait times with greater precision and accuracy is needed.

22. **Limitations – High rates of LGBTQ+ respondents is also a strength!**

RESPONSE: Thank you for your comment. We agree that the high rates of LGBTQ+ respondents is also a strength. We have now amended the discussion to highlight this point as a strength. Please see the edits highlighted in yellow in the revised manuscript.

23. **I see another strength of your study as including youth in the design of the survey and recruitment; I would consider writing about this as a strength in your discussion and in your conclusion talk about how youth should be involved in creating those best practices for addressing young peoples mental health needs**

RESPONSE: Thank you for your suggestion. We agree that including youth in the design of the survey and recruitment process is indeed a notable strength. We have revised the discussion and conclusion to highlight how youth involvement ensures that the research accurately reflects the experiences and preferences of young people, making the findings more relevant and applicable. Please see the edits highlighted in yellow in the revised manuscript.

Reviewer: 2

1. **Overall, the manuscript is very well written and presents a clear description of the study and findings. I have one major concern and two minor issues that need to be addressed before I can endorse the manuscript for publication.**

Most importantly, although the methods described to detect and remove fraudulent responses are comprehensive, the pattern of some of the data makes me question the validity of some of the included responses. The authors provide in the discussion some explanation for why the rates of LGBTQIA+ are so much higher than expected, this plus the high rates of non-binary and 'another identity' genders, and ATSI respondents make one question the data particularly as these groups were not targeted in recruitment. Also, the range of wait times for school counsellor goes up to 727 days.
Given this service, this does not seem a realistic time frame. Given this, perhaps the authors could conduct a sensitivity analysis whereby the analyses are repeated only for those participants who have provided responses to the open-ended questions that clearly indicate an authentic responder. If a similar pattern of results is found this would provide much more confidence in the study as a whole.

RESPONSE: We thank Reviewer 2 for their positive feedback regarding the manuscript's clarity and the presentation of the study and its findings.

We appreciate the concerns raised by Reviewer 2 regarding the demographic representation in our study and its potential implications for data validity. We have updated the **Limitations** section to clarify these points.

Notably, while the diversity rates observed in our sample may appear higher than those in the general adolescent population in Australia, they are consistent with findings from other mental health studies conducted at the Black Dog Institute and similar research contexts. For example, O'Dea et al. (2020) reported that 30.1% of adolescents (aged 12 to 14 years) participating in a trial evaluating a relationship-focussed mobile phone application, *WeClick*, identified as LGBTQIA+, and 3.6% identified as Aboriginal and/or Torres Strait Islander, despite these groups not being explicitly targeted during recruitment. Youth were recruited online, similar to our study. Similarly, another study examining the effects of the COVID-19 pandemic and the associated stages of lockdown on the characteristics of university student mental health app user subgroups in Australia also reported comparably high rates of participants identifying as LGBTQIA+ with the rates reported as 23%, 35%, and 38% at the three time points (Shvetcov et al., 2023). A more recent study evaluating a cognitive behavioural therapy smartphone application (ClearlyMe®) for reducing depressive symptoms in adolescents aged 12 to 17 years documented that 41.5% of adolescents identified as gender and/or sexuality diverse, with 5.3% identifying as non-binary and 3.2% reporting a different identity. In addition, 30 (5.3%) participants identified as Aboriginal and/or Torres Strait Islander.
Again, these groups were not specifically targeted in recruitment.

These patterns may reflect a heightened need for mental health support among gender and sexuality-diverse adolescents, as well as among Aboriginal and/or Torres Strait Islander youth. A recent study that reported current and comprehensive data about the status of adolescent mental health in Australia (N=6388, M=13.9 years) by Werner-Seidler et al. (2023) has also documented higher rates of mental illness in these populations, emphasising their vulnerability and likelihood of engaging in studies addressing mental health concerns. Additionally, the Black Dog Institute's allyship likely enhances its appeal to these groups. The Institute's reputation as a safe and supportive environment may explain why adolescents from diverse backgrounds are more inclined to participate in its research.

We have included the following references to support our findings:

1. O'Dea, B., Han, J., Batterham, P.J., Achilles, M.R., Calear, A.L., Werner-Seidler, A., Parker, B., Shand, F. and Christensen, H. (2020). A randomised controlled trial of a relationship-focussed mobile phone application for improving adolescents' mental health. Journal of Child Psychology and Psychiatry, 61: 899-913. https://doi.org/10.1111/jcpp.13294

2. Shvetcov, A., Whitton, A., Kasturi, S., Zheng, W-Y., Beames, J., Ibrahim, O., J., Han, Hoon, L., Mouzakis, K., Gupta, S., Venkatesh, S., Christensen, H., Newby, J. (2023). Machine learning identifies a COVID-19-specific phenotype in university students using a mental health app, Internet Interventions, 34. https://doi.org/10.1016/j.invent.2023.100666.

3. O'Dea, B., Li, S. H., Subotic-Kerry, M., Achilles, M. R., Mackinnon, A. J., Batterham., Christensen, H., Roberts, A., Nagendraprasad, K., Dudley, Z., Gillham, B., Werner-Seidler, A. (2020), The MobiliseMe study: A randomised controlled efficacy trial of a cognitive behavioural therapy smartphone application (ClearlyMe®) for reducing depressive symptoms in adolescents. https://www.medrxiv.org/content/10.1101/2024.11.17.24317363v1

4. Werner-Seidler A, Maston K, Calear AL, Batterham PJ, Larsen ME, Torok M, O'Dea B, Huckvale K, Beames JR, Brown L, Fujimoto H, Bartholomew A, Bal D, Schweizer S, Skinner SR, Steinbeck K, Ratcliffe J, Oei JL, Venkatesh S, Lingam R, Perry Y, Hudson JL, Boydell KM, Mackinnon A, Christensen H. (2023). The Future Proofing Study: Design, methods and baseline

characteristics of a prospective cohort study of the mental health of Australian adolescents. International Journal of Methods in Psychiatric Research, 32(3):e1954. doi: 10.1002/mpr.1954.

To ensure the validity of the data, we implemented a multi-faceted approach to detect and exclude fraudulent responses. One key criterion involved examining the content of free-response items, with all 375 participants included in this study providing at least one response to an open-ended question in the survey. This measure was integrated with several additional strategies to safeguard data integrity including fraud detection security measures embedded in the survey platform, examining the time taken to complete the survey and the time the survey was submitted, manual checks of IP addresses and email addresses, verifying geographic validity through postcode checks, identifying irregularities through patterns in survey responses, and analysing the content of free-response items for consistency and authenticity. To further enhance rigor, two researchers independently and manually reviewed the data to assess the validity of responses.

We thank Reviewer 2 for their comment regarding wait times and appreciate the opportunity to clarify this point. We acknowledge the potential for variability in responses to the wait-time questions, due to the phrasing of the following questions:

1. "*How long will you have waited between contacting this mental health professional or service and going to your first session? We understand that this can be hard to estimate, so just give it your best go*." or
2. "*From the time you or your family first contacted this service, how long did you have to wait before you had your first actual session? We understand that this can be hard to estimate, so just give it your best go.*")

These questions did not explicitly specify that participants should estimate wait times from the past year, which likely explains the responses exceeding 365 days.

To address concerns about potential overestimation and provide additional rigor, we conducted a re-analysis of the data, applying a stricter criterion by removing wait times exceeding one year. To align with our inclusion criteria (see our response below), we also removed values less than a week for adolescents who had previously waited more than one week in the past 12 months to access their first-ever treatment session.

Importantly, the re-analysis confirmed that the pattern of results examining the association between wait times and psychological distress remained consistent with our original findings. The re-analysis also confirmed the significant difference between wait times in metropolitan and regional areas to access a psychiatrist, consistent with our original findings. However, we identified an additional significant difference: wait times to access a paediatrician and a school counsellor were significantly longer in regional areas compared to metropolitan areas. Please see the edits highlighted in yellow in the revised manuscript.

Two minor issues:

a) The inclusion/exclusion criteria are a bit confusing. It seems that exclusion "(i) currently waiting for a follow-up treatment session with a mental health professional or service that they had accessed previously" contradicts the inclusion criteria of "or had previously waited (in the last 12 months) longer than one week to access their first session of mental health treatment with a mental health professional or service for symptoms of anxiety and/or depression".

RESPONSE: To enable participation from a greater number of adolescents and to include a greater range of wait times, we included two subgroups of participants (i) those who were currently waiting their first session, (ii) and those who had waited more than one week in

the past 12 months. To clarify this, we have amended the Participants section describing the inclusion and exclusion criteria:

“Adolescents were eligible to participate if they were aged 13-17 years old living in Australia and had sought treatment for anxiety and/or depression in the past 12 months. To enable greater exploration of wait times and participation among adolescents, we included two subgroups of participants (i) adolescents who were currently waiting to attend their first-ever session of mental health treatment (ii) adolescents who had waited more than one week in the past 12 months to access their first-ever session of treatment. Adolescents were excluded if they were (i) currently waiting for a follow-up treatment session with a mental health professional or service that they were not accessing for the first time, or (ii) currently waiting or previously waited for a treatment session that was unrelated to anxiety or depression.”

We hope these revisions provide greater clarity on the inclusion and exclusion criteria.

b) History of mental health is limited to anxiety/depression, this could be a limitation as many young people with these conditions have a history of other mental health and neurodevelopmental conditions which would influence their familiarity with accessing the mental health system and could impact on the findings.

RESPONSE: We agree that limiting the participant’s history of mental health to anxiety and/or depression is a limitation and have updated the limitations section to include the following:

“Finally, the current study did not measure the presence of co-occurring complexities that may have inflated wait times, such as the need for specialised mental health care (e.g. trauma, eating disorders, neurodivergence). Future work may benefit from greater attempts to understand how treatment seeking may be influenced by symptom severity, comorbidities, or additional psychosocial needs.”

VERSION 2 - REVIEW

**Reviewer**	**1**
**Name**	**Adams, Danielle R.**
**Affiliation**	**Washington University in St Louis**
**Date**	**15-Jan-2025**
**COI**	

The authors have done a great job of responding to my feedback. I believe the manuscript has been significantly strengthened and is ready for publication.
